# Case Report: Rare Homozygous *RNASEH1* Mutations Associated With Adult-Onset Mitochondrial Encephalomyopathy and Multiple Mitochondrial DNA Deletions

**DOI:** 10.3389/fgene.2022.906667

**Published:** 2022-05-31

**Authors:** Arianna Manini, Leonardo Caporali, Megi Meneri, Simona Zanotti, Daniela Piga, Ignazio Giuseppe Arena, Stefania Corti, Antonio Toscano, Giacomo Pietro Comi, Olimpia Musumeci, Valerio Carelli, Dario Ronchi

**Affiliations:** ^1^ Dino Ferrari Center, Department of Pathophysiology and Transplantation, University of Milan, Milan, Italy; ^2^ Istituto delle Scienze Neurologiche di Bologna, Programma di Neurogenetica, Bologna, Italy; ^3^ Foundation IRCCS Ca’ Granda Ospedale Maggiore Policlinico, Neurology Unit, Department of Neuroscience, Milan, Italy; ^4^ Foundation IRCCS Ca’ Granda Ospedale Maggiore Policlinico, Neuromuscular and Rare Diseases Unit, Department of Neuroscience, Milan, Italy; ^5^ Unit of Neurology and Neuromuscular disorders, Department of Clinical and Experimental Medicine, University of Messina, Messina, Italy; ^6^ Dipartimento di Scienze Biomediche e Neuromotorie (DIBINEM), University of Bologna, Bologna, Italy

**Keywords:** *RNASEH1*, ribonuclease H1, mitochondrial DNA, mtDNA maintenance disorders, myopathy, CPEO

## Abstract

Mitochondrial DNA (mtDNA) maintenance disorders embrace a broad range of clinical syndromes distinguished by the evidence of mtDNA depletion and/or deletions in affected tissues. Among the nuclear genes associated with mtDNA maintenance disorders, *RNASEH1* mutations produce a homogeneous phenotype, with progressive external ophthalmoplegia (PEO), ptosis, limb weakness, cerebellar ataxia, and dysphagia. The encoded enzyme, ribonuclease H1, is involved in mtDNA replication, whose impairment leads to an increase in replication intermediates resulting from mtDNA replication slowdown. Here, we describe two unrelated Italian probands (Patient 1 and Patient 2) affected by chronic PEO, ptosis, and muscle weakness. Cerebellar features and severe dysphagia requiring enteral feeding were observed in one patient. In both cases, muscle biopsy revealed diffuse mitochondrial abnormalities and multiple mtDNA deletions. A targeted next-generation sequencing analysis revealed the homozygous *RNASEH1* mutations c.129-3C>G and c.424G>A in patients 1 and 2, respectively. The c.129-3C>G substitution has never been described as disease-related and resulted in the loss of exon 2 in Patient 1 muscle *RNASEH1* transcript. Overall, we recommend implementing the use of high-throughput sequencing approaches in the clinical setting to reach genetic diagnosis in case of suspected presentations with impaired mtDNA homeostasis.

## Introduction

Mitochondrial DNA (mtDNA) maintenance disorders, which produce a variety of clinical presentations, including myopathy, progressive external ophthalmoparesis (PEO), ptosis, parkinsonism, bulbar dysfunction, and cerebellar features, originate from mutations in more than twenty-five nuclear genes involved in mtDNA homeostasis (Ahmed et al., 2015; Viscomi and Zeviani, 2017; Manini et al., 2022). Most of them encode for key proteins required for mtDNA replication and repair or for the local supply of deoxyribonucleotides used for mtDNA synthesis (Ahmed et al., 2015).

*RNASEH1*, located at chromosome 17p11.2, encodes the human ribonuclease H1 (RNase H1), an endonuclease that catalyzes the hydrolysis of RNA in RNA–DNA hybrids (Cerritelli and Crouch, 1998). RNase H1, which localizes both to the nucleus and to the mitochondria, takes part in mtDNA replication by processing long RNA primers of nascent strands, as proved by the significant decrease in mtDNA content in *Rnaseh1* ^*−/−*^ embryos, with developmental arrest and massive apoptosis (Cerritelli et al., 2003). The pivotal role of RNase H1 in mtDNA transcription and synthesis and mitochondrial function was confirmed by Lima et al. (2016) in a liver-specific RNase H1 knockout mice expressing an inactive RNase H1 mutant. Furthermore, RNase H1 is involved in the clearance of R-loops, produced during mtDNA transcription, which can impair mtDNA structural integrity (Lima et al., 2016; Holt, 2019).

To date, six *RNASEH1* mutations have been reported in 16 patients presenting with progressive external ophthalmoplegia (PEO), ptosis, limb weakness, cerebellar ataxia, and dysphagia and displaying accumulation of multiple mtDNA deletions and increased 7S DNA levels in muscle (Lieber et al., 2013; Reyes et al., 2015; Bugiardini et al., 2017; Sachdev et al., 2018; Carreño-Gago et al., 2019). The 7S DNA represents an mtDNA replication intermediate that is scarcely processed by MGME1, thus resulting in aborted replication (Kornblum et al., 2013).

Here, we report two Italian patients with adult-onset mitochondrial encephalomyopathy and multiple mtDNA deletions, each one harboring a homozygous *RNASEH1* mutation: c.129-3C>G and c.424G>A. The c.129-3C>G variant, which causes the loss of exon 2 at the transcript level, has never been reported before in patients with mtDNA instability.

## Case Description

### Patient 1

The patient is a 60-year-old Italian man, born to healthy, non-consanguineous parents after uncomplicated pregnancy and delivery. He had normal psychomotor development, and his medical history includes only slight hypertension.

**FIGURE 1:**
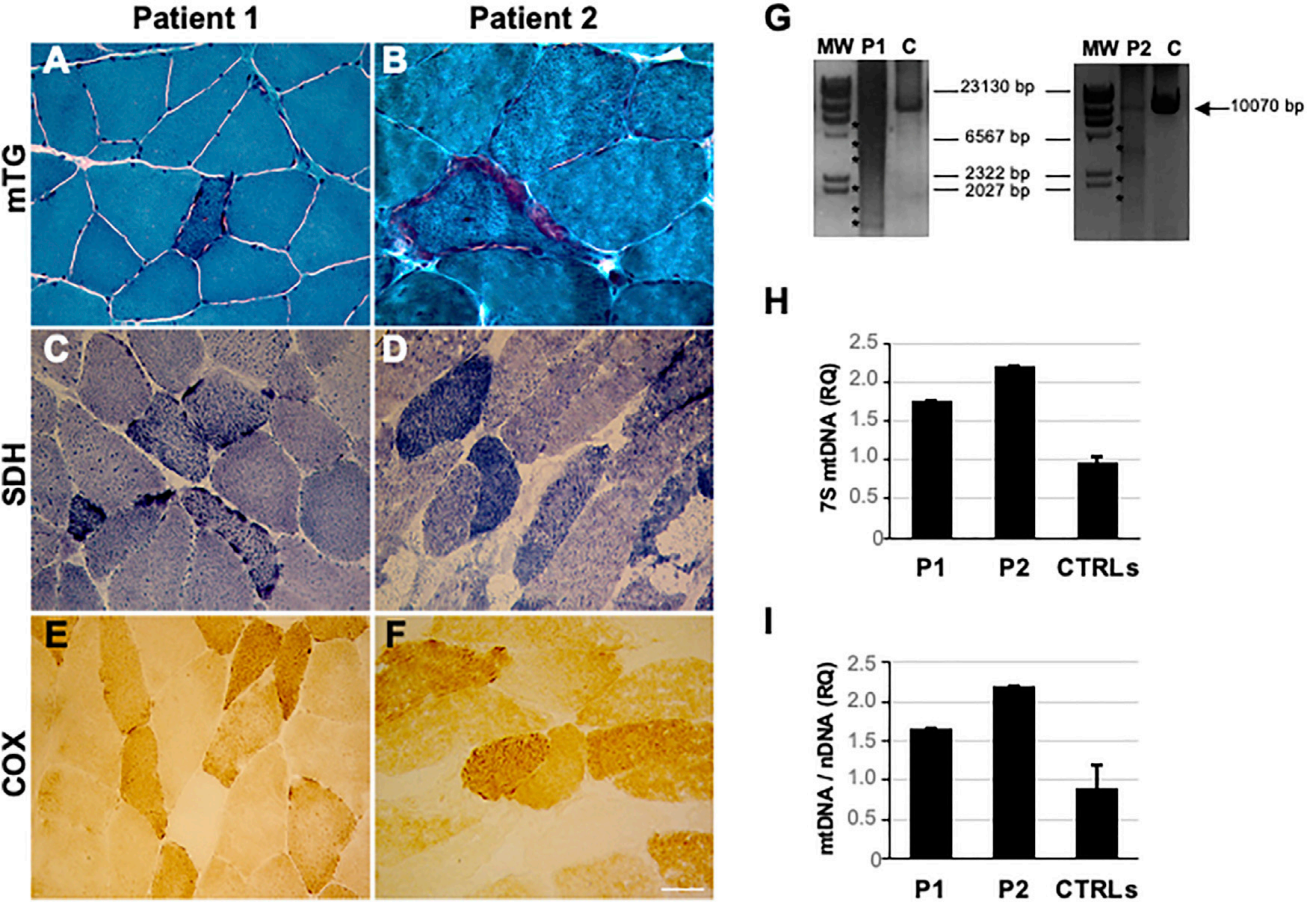
Histological and mtDNA studies in *RNASEH1*-mutated patients (P1 and P2). **(A, B)** Modified Gomori trichrome (MGT) showing the presence of ragged red fibers in patients’ muscle sections. **(C, D)** SDH staining showed the presence of SDH-positive fibers. **(E, F)** COX histochemical reaction documenting the presence of COX-negative fibers. Magnification 40x. Scale bar 50 μm. **(G)** Long-range PCR analysis of mitochondrial DNA obtained from the probands’ muscle biopsy (P1 and P2) and healthy controls (C). Asterisks indicate multiple bands corresponding to multiple mtDNA deletions. The black arrow indicates the expected size of the wild-type PCR amplicon (10.5 kB: FOR5635-RC16135). The sizes of the bands of the DNA molecular weight marker II (MW, Roche) are indicated. **(H)** Histogram showing levels of muscle 7S mtDNA in patients compared to healthy controls (n = 14). The ratio (RQ) indicates 7S mtDNA levels with respect to the total number of mitochondrial genomes amplified by quantitative PCR. Error bars indicate standard deviations. **(I)** Histogram showing muscle mtDNA content in the proband compared to healthy controls (n = 27). mtDNA quantification, normalized to nuclear DNA (nDNA) content, was performed by quantitative PCR. Error bars indicate standard deviations.

Since the age of 40, he developed progressive muscular weakness, impairment of eye movements, ptosis, and hypoacusis. During disease progression, signs of cerebellar involvement and ataxia were reported. Electromyography (EMG) findings were consistent with a myopathic picture. Brain magnetic resonance imaging (MRI) revealed non-specific, diffuse white matter hyperintensities. Biopsy of the deltoid demonstrated considerable fiber size variation with severely hypotrophic angulated fibers (mainly type 1), splitting fibers, central nuclei, and mitochondrial dysfunction features including diffuse ragged-red fibers (RRFs), succinate dehydrogenase (SDH)-positive, and cytochrome *c* oxidase (COX)-negative fibers (17.70%, calculated on 10 non-overlapping fields at 20x magnification) (Figures 1A, C, E).

After the diagnosis of mitochondrial encephalomyopathy, therapeutic attempts with idebenone, coenzyme Q, riboflavin, and vitamin E were made, with no clinical benefit.

At 52 years of age, the patient was hospitalized to place a percutaneous endoscopic gastrostomy (PEG) due to severe dysphagia. During hospitalization, creatine phosphokinase (CK) levels reached 1,298 IU/L (normal value < 200). Electrocardiogram and echocardiogram were normal.

The last clinical examination, performed eight years ago, revealed bilateral ptosis, bilateral ophthalmoplegia, marked dysphonia, dysarthria, and dysphagia of both solid food and liquids. Mild proximal muscle weakness, more pronounced in the lower limbs, dysmetria, and extensor plantar responses were present bilaterally. Deep tendon reflexes (DTRs) were diffusely reduced. The patient showed unsteadiness in the standing position. He was able to walk only with support, revealing a spastic gait.

### Patient 2

The patient is a 70-year-old Italian man, with no family history of neuromuscular disorders. At 47 years of age, he developed progressive muscle weakness, limitation in ocular motility, and gait unsteadiness. At 53 years, he was hospitalized because of the progression of symptoms. Neurological examination revealed bilateral eyelid ptosis with ophthalmoplegia, ataxic gait, and dysarthria with muscle weakness involving mainly upper limbs, absence of deep tendon reflexes, dysmetria, bilateral Babinski sign, and reduced vibratory sense in lower limbs. The nerve conduction study (NCS) showed a severe sensorimotor axonal neuropathy. Electrocardiogram and echocardiogram were normal. The electroencephalogram was unremarkable. Brain MRI showed cerebellar atrophy. Muscle biopsy revealed significant fiber size variation, necrosis and regenerating fibers, fiber type grouping at ATPase pH 4.3 (predominantly 2A and, to a lesser extent, 2C; rarely 2B), increased lipid storage, and mitochondrial dysfunction features including some RRFs and several COX-negative fibers (36.07%) (Figures 1B, D, F). Biopsy of the right sural nerve showed severe loss of myelinated fibers with few clusters of regeneration.

At the last clinical examination, performed 10 years ago, the patient was wheelchair-bound and showed bilateral ptosis and ophthalmoplegia, dysphonia, dysarthria, dysphagia, proximal muscle weakness of four limbs, dysmetria, and absence of DTRs.

**FIGURE 2:**
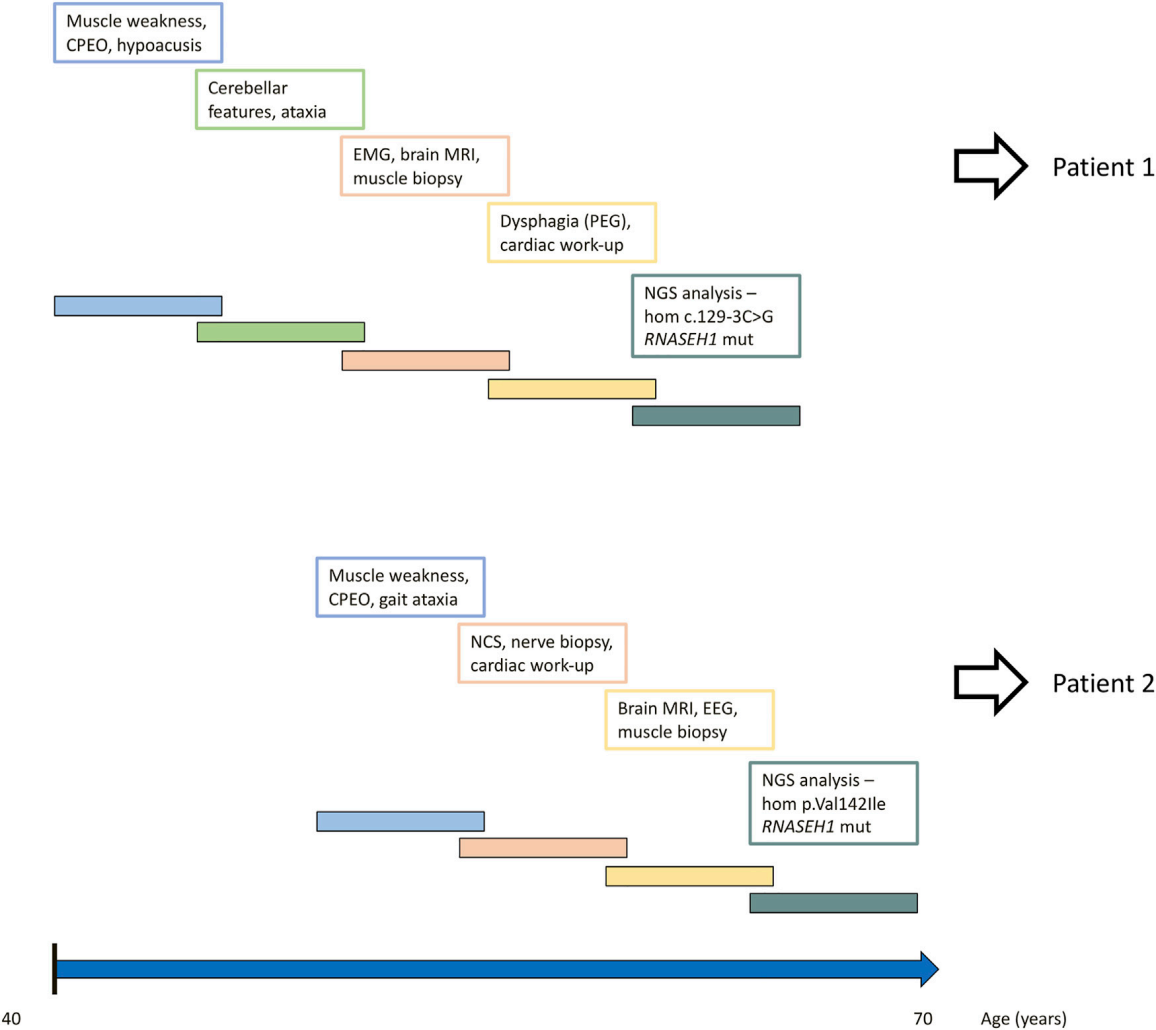
Timeline of relevant clinical signs and symptoms and of diagnostic testing performed during disease progression of both patients.

The timeline of relevant clinical signs and symptoms and of diagnostic testing performed during the disease progression of both patients is reported in Figure 2.

## Results

Long-range polymerase chain reaction (PCR), performed as previously described (Ronchi et al., 2012), revealed multiple mtDNA deletions in patients’ muscle (Figure 1G), quantified by quantitative PCR (12 and 28% for patients 1 and 2, respectively). Total mtDNA and 7S mtDNA levels were investigated by quantitative PCR in muscle. Determinations were performed in triplicate using fluorescent TaqMan assays (Reyes et al., 2015). Levels of 7S mtDNA, the third strand of the mtDNA displacement loop, were significantly higher in the probands’ muscle than those in controls, suggesting aborted mtDNA replication (Figure 1H). Higher mtDNA content was found in mutated specimens, likely reflecting mitochondrial proliferation (Figure 1I).

Both the probands underwent a next-generation sequencing (NGS) analysis by a panel of 24 genes associated with mtDNA maintenance disorders (Supplementary Table S1). The library was generated by using a 250-bp amplicon-based approach (TruSeq Custom Amplicon, Illumina) and sequenced on a MiSeq instrument (Illumina). Reads were aligned to the human genome (assembly hg19), and the identified variants were annotated (ANNOVAR) and filtered, focusing on rare variants (≤0.5% in public databases), causing changes potentially damaging for the protein function [Combined Annotation Dependent Depletion (CADD) and deleterious annotation of genetic variants using neural networks (DANN)]. The variants identified were checked on the BAM files using IGV software (https://software.broadinstitute.org/software/igv/).

**FIGURE 3:**
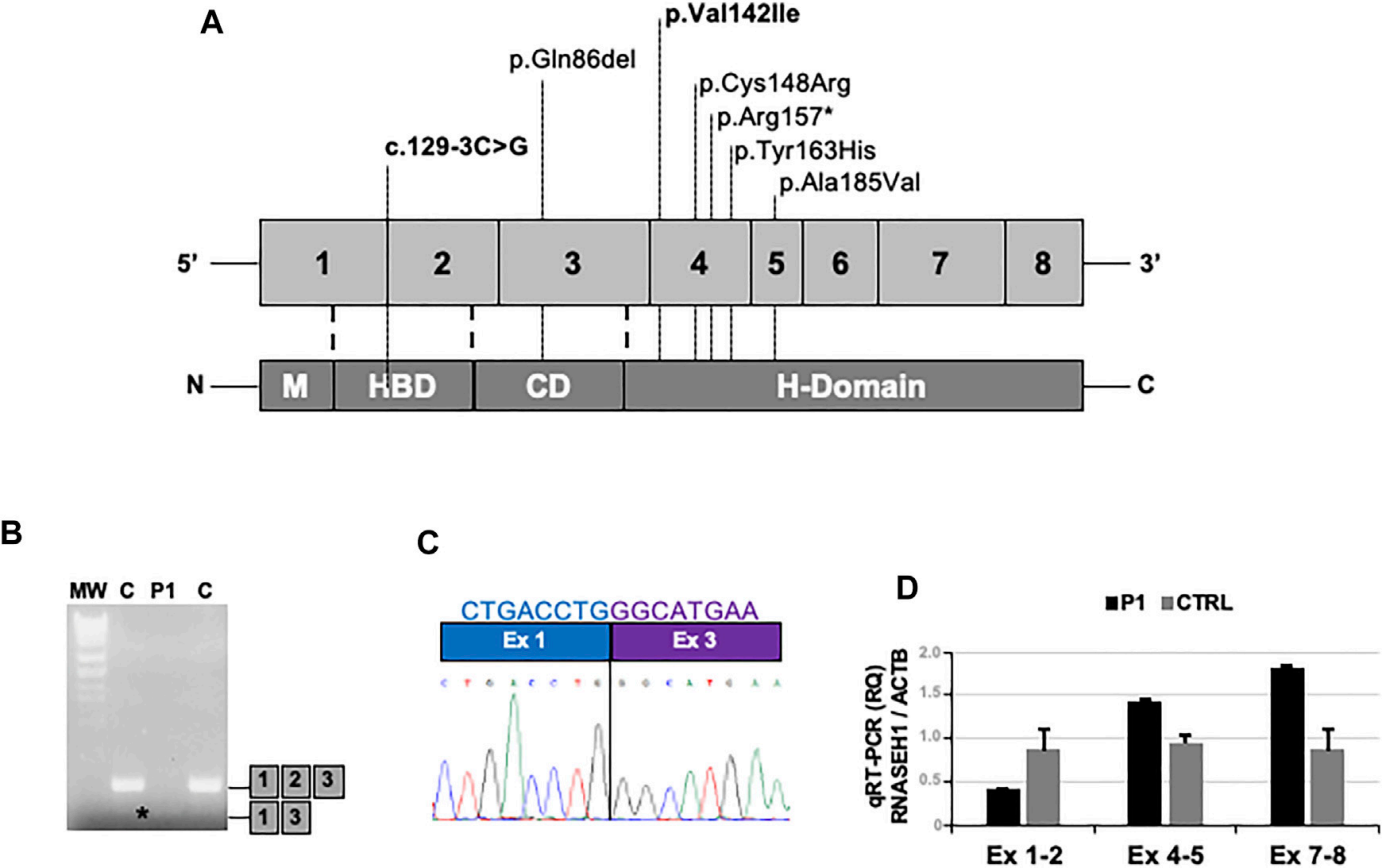
**–** Molecular studies. **(A)** Schematic representation of the *RNASEH1* gene and protein structure, showing the location of all the pathogenic variants described so far; those detected in the present work are highlighted in bold. **(B)** Gel electrophoresis of RT-PCR amplicons encompassing exons 1 and 3 in muscles of the proband (P1) and controls (C). Asterisk indicates the presence of an abnormal band in P1. **(C)** Sequence electropherogram showing the loss of exon 2 in the proband’s RT-PCR amplicon. **(D)** Quantitative RT-PCR analysis of *RNASEH1* transcript showing the selective reduction of the signal of the probe targeting exon 1–2 junction in the proband’s muscle compared to controls.

In Patient 1, NGS analysis identified the chr2:3552308G/C homozygous change corresponding to c.129-3C>G mutation in *RNASEH1* (NM_002936.6) (Figure 3A). In Patient 2, the homozygous c.424G>A (p.Val142Ile) *RNASEH1* was observed (Figure 3A). This variant has already been reported in independent cases of adult-onset mitochondrial encephalomyopathy with multiple mitochondrial DNA deletions, as summarized in Table 1 (Lieber et al., 2013; Reyes et al., 2015; Bugiardini et al., 2017; Sachdev et al., 2018). Both variants were confirmed by Sanger sequencing. DNA from relatives was not available for segregation analysis.

**TABLE 1:** List of *RNASEH1*-mutated patients described so far.

Ref	Pt	AO	Allele 1	Allele 2	Clinical finding	CT/MRI	EMG/NCS	Muscle biopsy	mtDNA dels
Lieber et al. (2013)	1,019	44	c.424G>A p.Val142Ile	c.442T>C p.Cys148Arg	PEO, ptosis, ataxia, fatigue, and dysphagia	NA	NA	RRFs and COX-deficient fibers	NA
Reyes et al. (2015)	S1	20	c.424G>A p.Val142Ile	c.469C>T p.Arg157*	PEO, ptosis, dysphagia, dysarthria, dysphonia, muscle pain, exercise intolerance, respiratory and lower limb weakness, and ataxia	Cerebellar and brain stem atrophy	Mild demyelinating motor neuropathy and myopathy	RRFs and COX-deficient fibers	Y
	S2	23	c.424G>A p.Val142Ile	c.554C>T p.Ala185Val	PEO, ptosis, fatigue, limb and axial weakness, head drop, pyramidal signs, dysphagia, reduced visual acuity, and cerebellar signs	NA	Mild neurogenic features	RRFs and COX-deficient fibers	Y
	S3	ND	c.424G>A p.Val142Ile	c.424G>A p.Val142Ile	PEO, dysphagia, and respiratory impairment	NA	NA	NA	Y
	S4	ND	c.424G>A p.Val142Ile	c.424G>A p.Val142Ile	PEO, dysphagia, and respiratory impairment	NA	NA	NA	Y
	S5	45	c.424G>A p.Val142Ile	c.424G>A p.Val142Ile	PEO, ptosis, dysphonia, dysphagia, pyramidal signs, cerebellar signs, and cognitive impairment	Cerebellar and cortical atrophy and white matter hyperintensities	Neuropathy	RRFs and COX-deficient fibers	NA
	S6	40	c.424G>A p.Val142Ile	c.424G>A p.Val142Ile	PEO, gait instability, severe dysphagia, and respiratory impairment	NA	NA	RRFs and COX-deficient fibers	Y
Bugiardini et al. (2017)	A-III.8	33	c.424G>A p.Val142Ile	c.424G>A p.Val142Ile	PEO, ptosis, facial weakness, and proximal muscle weakness	Normal	Myopathy	RRFs and COX-deficient fibers	Y
	A-III.9	32	c.424G>A p.Val142Ile	c.424G>A p.Val142Ile	PEO and ptosis	NA	NA	RRFs	NA
	A-III.10	ND	c.424G>A p.Val142Ile	c.424G>A p.Val142Ile	PEO and ptosis	NA	NA	NA	NA
	A-III.11	ND	c.424G>A p.Val142Ile	c.424G>A p.Val142Ile	PEO and ptosis	NA	NA	NA	NA
	B-II.1	36	c.424G>A p.Val142Ile	c.424G>A p.Val142Ile	PEO, ptosis, ataxia, and facial and proximal muscle weakness	Cerebral and cerebellar atrophy	NA	SDH-positive/COX-deficient fibers	Y
	B-II.8	33	c.424G>A p.Val142Ile	c.424G>A p.Val142Ile	PEO, ptosis, dysarthria, ataxia, and facial and proximal muscle weakness	Cerebral and cerebellar atrophy	Sensorimotor neuropathy and myopathy	RRFs, SDH-positive/COX-deficient fibers, and neurogenic changes.	Y
Bugiardini et al. (2017); Sachdev et al. (2018)	C-II.1	13	c.424G>A p.Val142Ile	c.442T>C p.Cys148Arg	PEO, ptosis, reduced visual acuity, proximal muscle weakness, ataxia, and type II diabetes	NA	Sensory neuropathy	RRFs	Y
Carreño-Gago et al. (2019)	P1	53	c.258_260del p.Gln86del	c.487T>C p.Tyr163His	PEO, ptosis, muscle weakness, dysphagia, dysarthria, and respiratory impairment	NA	NA	RRFs and COX-deficient fibers	Y
*Current*	P1	40	c.129-3C>G	c.129-3C>G	PEO, ptosis, progressive muscle weakness, peripheral neuropathy, cerebellar signs, ataxia, dysphagia, impaired hearing, and stroke-like episodes	White matter hyperintensities	Myopathic changes	RRFs and SDH-positive/COX-deficient fibers	Y
	P2	47	c.424G>A p.Val142Ile	c.424G>A p.Val142Ile	PEO, ptosis, progressive muscle weakness, peripheral neuropathy, ataxic gait, dysphonia, dysarthria, and dysphagia	Cerebellar atrophy	Severe axonal neuropathy	RRFs and COX-deficient fibers	Y

Ref: reference; Pt: patient; AO: age at onset; CT: computed tomography; MRI: magnetic resonance imaging; EMG: electromyography; mtDNA: mitochondrial DNA; del: deletions; PEO: progressive external ophthalmoplegia; RRFs: ragged-red fibers; COX: cytochrome *c* oxidase; NA: not available; Y: yes; SDH: succinate dehydrogenase.

The c.129-3C>G variant has never been found in public databases. As it is predicted to alter the acceptor splice site of exon 2 by Alternative Splice Site Predictor (ASSP) and NetGene2 tools, complementary DNA (cDNA) was obtained from muscle-extracted RNA by using the Maxima reverse transcriptase (Life Technologies) and analyzed. Reverse transcriptase (RT)-PCR amplicon encompassing exons 1 and 3 showed reduced molecular weight (Figure 3B). The loss of exon 2 in the patient’s muscle was demonstrated by Sanger sequencing (Figure 3C) and supported by quantitative RT-PCR analysis (Figure 3D) performed by using TaqMan assays (Life Technologies): Hs01108219_g1 (exon junction 1–2), Hs01108222_g1 (exon junction 4–5), Hs00268000_m1 (exon junction 7–8), and Hs99999903_m1 (ACTB, housekeeping).

## Discussion

We reported two novel *RNASEH1*-mutated patients affected by chronic PEO, ptosis, and moderate muscle weakness, involving predominantly lower limbs. Both showed cerebellar features and severe dysphagia requiring enteral feeding by PEG in one of them. While in Patient 1 both neurophysiological and histological findings were consistent with a myopathic process, nerve conduction studies in Patient 2 revealed a severe axonal neuropathy, with loss of myelinated fibers at nerve biopsy. Muscle biopsy showed mitochondrial abnormalities, with diffuse RRFs, SDH-positive, COX-negative fibers, and multiple mtDNA deletions accumulating in muscle. Overall, clinical findings and diagnostic testing pointed toward a mitochondrial encephalomyopathy belonging to the spectrum of mtDNA maintenance disorders. A targeted gene panel approach unraveled the homozygous c.129-3C>G in Patient 1 and c.424G>A in Patient 2.

Over the last years, *RNASEH1* mutations have been associated with a mild-to-moderate form of adult-onset encephalomyopathy (Table 1) (Reyes et al., 2015; Bugiardini et al., 2017; Sachdev et al., 2018; Carreño-Gago et al., 2019). In *RNASEH1*-related disease, the clinical picture is dominated by PEO, dysphagia, dysarthria, respiratory impairment, and cerebellar signs (Reyes et al., 2015; Bugiardini et al., 2017). While PEO (with or without ptosis) is a common clinical finding in adult presentations of mtDNA maintenance disorders, the additional clinical symptoms frequently observed in *RNASEH1*-mutated patients have been previously observed separately in distinct genetic forms featuring altered mtDNA homeostasis: dysphagia in adult-onset *DGUOK* patients, dysarthria in adult patients with *POLG* and *TK2* mutations, progressive respiratory impairment in *MGME1*-mutated patients, and cerebellar signs in late-onset presentations due to *RRM2B* defects (Ahmed et al., 2015; Viscomi and Zeviani, 2017).

The clinical phenotype of Patient 2 partially overlaps with findings in other patients presenting the homozygous p.Val142Ile mutation reported in the literature (Table 1). Age at onset of homozygous p.Val142Ile carriers ranges from 32 to 47 years. Conversely, patients, whose p.Val142Ile segregates in trans with a second RNASEH1 variant, show a more variable age at onset, ranging from 13 to 44 years, thus suggesting that the second mutation might deeply influence the development and progression of the disorder. Despite showing a late-onset disease, instead, our patient presenting the homozygous c.129-3C>G variant developed a severe phenotype, with prevalent bulbar signs, including PEG-requiring dysphagia, in addition to PEO, ptosis, progressive muscle weakness, peripheral neuropathy, and cerebellar features. Additional cases of patients harboring this novel mutation are required to drive conclusions on the specific genotype–phenotype correlation.

The c.129-3C>G has never been described as disease-related. Although DNA from parents was not available for co-segregation analysis, their asymptomatic status supports the previously described autosomal recessive inheritance of *RNASEH1*-related encephalomyopathy (Reyes et al., 2015; Bugiardini et al., 2017). As proved by cDNA Sanger sequencing and quantitative RT-PCR analysis, the c.129-3C>G mutation produces loss of exon 2, which encodes for part of the hybrid-binding domain (HBD), and for part of the flexible connection domain (CD) (Figure 3A) (Carreño-Gago et al., 2019). The HBD takes part in the identification of DNA–RNA hybrids, whereas the CD binds the HBD to the C-terminal catalytic domain (Reyes et al., 2015; Carreño-Gago et al., 2019). All the previously described *RNASEH1* mutations, including the p.Val142Ile, are located at the catalytic domain, except for c.258_260del, p.Gln86del, which maps to the CD (Figure 3A) (Lieber et al., 2013; Reyes et al., 2015; Bugiardini et al., 2017; Sachdev et al., 2018; Carreño-Gago et al., 2019). Therefore, our report expands the spectrum of mutational hot spots within *RNASEH1*.

According to the American College of Medical Genetics and Genomics (ACMG) guidelines for the interpretation of sequence variants, the c.129-3C>G variant can be classified as “pathogenic” because it meets the pathogenicity criteria PSV1, PM2, PP3, and PP4 (Richards et al., 2015). Carreño-Gago et al. (2019) described a patient who harbored two likely pathogenic homozygous mutations in *RNASEH1*, namely, the c.258_260del, p.(Gln86del) and the c.487T>C, p.(Tyr163His). By studying patient’s fibroblasts, the authors demonstrated that at least one of the *RNASEH1* variants affects protein stability, as RNase H1 levels in mitochondria were markedly reduced (Carreño-Gago et al., 2019). Furthermore, an *in vitro* functional assay showed absence of residual RNase H1 activity associated with both mutated recombinant proteins (Carreño-Gago et al., 2019). Similarly, the nonsense c.469C>T, p.Arg157* mutation reported by Reyes et al. (2015), which maps to the catalytic domain, resulted in significantly reduced transcript levels and protein instability. The mutation found in Patient 1 is a splice-site variant with a proven functional impact on the transcript. Taken together, these findings suggest that the common underlying pathogenic mechanism might be of loss-of-function.

In addition, Reyes et al. (2015) described an increase in mtDNA replication intermediates resulting from mtDNA replication slowdown. RNase H1, indeed, is involved in the processing of long RNA primers of nascent strands (Reyes et al., 2015). When RNase H1 activity is impaired, the mtDNA replication intermediates increase, thus hindering the replication process itself (Reyes et al., 2015). Consistently with the findings of Reyes et al. (2015), we detected increased 7S levels normalized to total mtDNA content in our patients.

In summary, this report confirms the pathogenic role of the p.Val142Ile *RNASEH1* mutation and expands the genetic background of *RNASEH1*-related encephalomyopathy. As demonstrated by this report, the implementation of high-throughput sequencing approaches is warranted to reach genetic diagnosis in the clinical setting, especially when heterogeneous disorders are suspected, including clinical syndromes due to impaired mtDNA maintenance.

## Data Availability

The datasets for this article are not publicly available due to concerns regarding participant/patient anonymity. Requests to access the datasets should be directed to the corresponding authors.

## References

[B1] Ahmed N. Ronchi D. Comi G. (2015). Genes and Pathways Involved in Adult Onset Disorders Featuring Muscle Mitochondrial DNA Instability. Ijms 16, 18054–18076. 10.3390/ijms160818054 26251896 PMC4581235

[B2] Bugiardini E. Poole O. V. Manole A. Pittman A. M. Horga A. Hargreaves I. (2017). Clinicopathologic and Molecular Spectrum of RNASEH1-Related Mitochondrial Disease. Neurol. Genet. 3, e149. 10.1212/NXG.0000000000000149 28508084 PMC5413961

[B3] Carreño-Gago L. Blázquez-Bermejo C. Díaz-Manera J. Cámara Y. Gallardo E. Martí R. (2019). Identification and Characterization of New RNASEH1 Mutations Associated with PEO Syndrome and Multiple Mitochondrial DNA Deletions. Front. Genet. 10. 10.3389/fgene.2019.00576 PMC6588129 31258551

[B4] Cerritelli S. M. Crouch R. J. (1998). Cloning, Expression, and Mapping of Ribonucleases H of Human and Mouse Related to Bacterial RNase HI. Genomics 53, 300–307. 10.1006/geno.1998.5497 9799596

[B5] Cerritelli S. M. Frolova E. G. Feng C. Grinberg A. Love P. E. Crouch R. J. (2003). Failure to Produce Mitochondrial DNA Results in Embryonic Lethality in Rnaseh1 Null Mice. Mol. Cell 11, 807–815. 10.1016/S1097-2765(03)00088-1 12667461

[B6] Holt I. J. (2019). The Jekyll and Hyde Character of RNase H1 and its Multiple Roles in Mitochondrial DNA Metabolism. DNA Repair 84, 102630. 10.1016/j.dnarep.2019.06.001 31178343

[B7] Kornblum C. Nicholls T. J. Haack T. B. Schöler S. Peeva V. Danhauser K. (2013). Loss-of-function Mutations in MGME1 Impair mtDNA Replication and Cause Multisystemic Mitochondrial Disease. Nat. Genet. 45, 214–219. 10.1038/ng.2501 23313956 PMC3678843

[B8] Lieber D. S. Calvo S. E. Shanahan K. Slate N. G. Liu S. Hershman S. G. (2013). Targeted Exome Sequencing of Suspected Mitochondrial Disorders. Neurology 80, 1762–1770. 10.1212/WNL.0b013e3182918c40 23596069 PMC3719425

[B9] Lima W. F. Murray H. M. Damle S. S. Hart C. E. Hung G. De Hoyos C. L. (2016). ViableRNaseH1knockout Mice Show RNaseH1 Is Essential for R Loop Processing, Mitochondrial and Liver Function. Nucleic Acids Res. 44, 5299–5312. 10.1093/nar/gkw350 27131367 PMC4914116

[B10] Manini A. Abati E. Comi G. P. Pietro, Corti S. Corti D. (2022). Mitochondrial DNA Homeostasis Impairment and Dopaminergic Dysfunction: A Trembling Balance. Ageing Res. Rev. 76, 101578. 10.1016/j.arr.2022.101578 35114397

[B11] Reyes A. Melchionda L. Nasca A. Carrara F. Lamantea E. Zanolini A. (2015). RNASEH1 Mutations Impair mtDNA Replication and Cause Adult-Onset Mitochondrial Encephalomyopathy. Am. J. Hum. Genet. 97, 186–193. 10.1016/j.ajhg.2015.05.013 26094573 PMC4572567

[B12] Richards S. Aziz N. Bale S. Bick D. Das S. Gastier-Foster J. (2015). Standards and Guidelines for the Interpretation of Sequence Variants: a Joint Consensus Recommendation of the American College of Medical Genetics and Genomics and the Association for Molecular Pathology. Genet. Med. 17, 405–424. 10.1038/gim.2015.30 25741868 PMC4544753

[B13] Ronchi D. Garone C. Bordoni A. Gutierrez Rios P. Calvo S. E. Ripolone M. (2012). Next-generation Sequencing Reveals DGUOK Mutations in Adult Patients with Mitochondrial DNA Multiple Deletions. Brain 135, 3404–3415. 10.1093/brain/aws258 23043144 PMC3501975

[B14] Sachdev A. Fratter C. McMullan T. F. W. (2018). Novel Mutation in the RNASEH1 Gene in a Chronic Progressive External Ophthalmoplegia Patient. Can. J. Ophthalmol. 53, e203–e205. 10.1016/j.jcjo.2018.01.005 30340744

[B15] Viscomi C. Zeviani M. (2017). MtDNA-maintenance Defects: Syndromes and Genes. J. Inherit. Metab. Dis. 40, 587–599. 10.1007/s10545-017-0027-5 28324239 PMC5500664

