# Supplementary material for: Case Report: Rare Homozygous RNASEH1 Mutations Associated With Adult-Onset Mitochondrial Encephalomyopathy and Multiple Mitochondrial DNA Deletions
Source: Front Genet. 2022 May 31;13:906667. doi: 10.3389/fgene.2022.906667 (PMC9194440; doi:10.3389/fgene.2022.906667)
Supplement: Supplementary file 1 [file Table1.DOCX]

**Supplementary Table 1** – List of genes associated with mtDNA maintenance disorders included in our NGS panel.

| **Gene** | **Inheritance** | **OMIM** | **Locus** | **Refseq** |
| --- | --- | --- | --- | --- |
| AFG3L2 | AD | *604581, #610246 | 18p11.21 | NM_006796.3 |
| CHCHD10 | AD | *615903, #615911 | 22q11.23 | NM_001301339.2 |
| DGUOK | AR | *601465, #617070 | 2p13.1 | NM_080916.3 |
| DNA2 | AD | *601810, # 615156 | 10q21.3 | NM_001080449.3 |
| DNM1L | AD, AR | *603850 | 12p11.21 | NM_012062.5 |
| FBXL4 | AR | *605654, #615471 | 6q16.1-q16.2 | NM_012160.4 |
| GFER | AR | *600924, #613076 | 16p13.3 | NM_005262.3 |
| MFN1 | unknown | *608506 | 3q26.33 | NM_001206614.1 |
| MFN2 | AD | *608507 | 1p36.22 | NM_014874.4 |
| MGME1 | AR | *615076 | 20p11.23 | NM_001310338.2 |
| MPV17 | AR | *137960 | 2p23.3 | NM_002437.5 |
| OPA1 | AD | *605290, #125250 | 3q29 | NM_130837.3 |
| POLG | AD, AR | *174763, #157640, #258450 | 15q26.1 | NM_002693.3 |
| POLG2 | AD | *604983, #610131 | 17q23.3 | NM_007215.4 |
| RNASEH1 | AR | *604123, #616479 | 2p25.3 | NM_002936.6 |
| RRM2B | AR | *604712, #613077 | 8q22.3 | NM_015713.5 |
| SLC25A4 | AD | *103220, #609283 | 4q35.1 | NM_001151.4 |
| SPG7 | AR | *602783 | 16q24.3 | NM_001363850.1 |
| SSBP1 | AD | *600439 | 7q34 | NM_001256510.1 |
| SUCLA2 | AR | *603921, #612073 | 13q14.2 | NM_003850.3 |
| SUCLG1 | AR | *611224, #245400 | 2p11.2 | NM_003849.4 |
| TK2 | AR | *188250, #617069 | 16q21 | NM_004614.5 |
| TWNK | AD | *606075, #609286 | 10q24.31 | NM_021830.5 |
| TYMP | AR | *131222, #603041 | 22q13.33 | NM_001953.5 |

AD=Autosomal Dominant, AR=Autosomal recessive.
